# A machine vision system for tracking population behavior of zooplankton in small-scale experiments: a case study on salmon lice (*Lepeophtheirus salmonis* Krøyer, 1838) copepodite population responses to different light stimuli

**DOI:** 10.1242/bio.050724

**Published:** 2020-06-25

**Authors:** Bjarne Kvæstad, Trond Nordtug, Andreas Hagemann

**Affiliations:** SINTEF Ocean, Environment and New Resources, Brattørkaia 17C, NO-7010 Trondheim, Norway

**Keywords:** Machine vision, Behavior study, Zooplankton

## Abstract

To achieve efficient and preventive measures against salmon lice (*Lepeophtheirus salmonis* Krøyer, 1838) infestation, a better understanding of behavioral patterns of the planktonic life stages is key. To investigate light responses in *L. salmonis* copepodites, a non-intrusive experimental system was designed to measure behavioral responses in a 12.5-l volume using machine vision technology and methodology. The experimental system successfully tracked the collective movement patterns of the sea lice population during exposure to different light stimuli emitted from alternating zones in the system. This system could further be used to study behavioral responses to different physical cues of various developmental stages of sea lice or other zooplankton.

## Abstract (precis)

**Summary:** A suggested method for tracking large populations (*n*=200) of small (<1 mm length) zooplankton for behavior study when exposed to stimuli.

## INTRODUCTION

Salmon lice (*Lepeophtheirus salmonis* Krøyer, 1838) are considered a threat for wild salmon populations and constitute a major economic cost to salmon farmers (Costello, 2009). Identifying behavioral patterns of *L. salmonis* in response to environmental and physical cues is key to understanding the behavioral ecology of the salmon louse, and to achieve efficient preventive measures against salmon lice infestation. Planktonic sea lice dispersal models can be used to predict infection pressures linked to releases of nauplii from point sources (Amundrud and Murray, 2009; Kristoffersen et al., 2014; Myksvoll et al., 2018). Such models can use behavioral data from small-scale experiments to improve the predictive accuracy of the (infectious) copepodite stages of salmon lice such as light response in the context of underwater lighting around salmon sea cages.

Conducting real-time observational studies on zooplankton organisms in experimental systems without interfering with the organism is a complex task due to the organism's small size and rapid and sometimes erratic movement pattern (Solvang and Hagemann, 2018). Induced by the strong economic interests of the salmon farming industry to combat sea lice infestation, several experimental systems have been designed in the past few decades to study *L. salmonis* behavior in relation to external cues and host interactions, both in enclosures at sea (Heuch et al., 1995, 1996; Hevrøy et al., 2003) and in laboratory systems (Gravil, 1996; Aarseth and Schram, 1999; Flamarique et al., 2000; Bailey et al., 2006; Fields et al., 2018; Solvang and Hagemann, 2018). Simultaneously tracking high numbers of zooplankton in larger water volumes in such experimental systems has historically been challenging due to the small size of the organisms, which demands high image resolution, and this has so far limited either the measurement area or the sample frequency.

Motivated by a study on light preferences for *L. salmonis* copepodites (Nordtug et al., submitted), a non-intrusive experimental system was designed to measure behavioral responses in terms of horizontal population displacements to light stimuli in large populations. The aim of our experimental system was to push the limits for water volume and extension of the measurement area while being able to track horizontal movement of a large population of *L. salmonis* copepodites without blind zones. In this study, the experimental setup where these requirements are fulfilled involved using machine vision technology.

## RESULTS AND DISCUSSION

The experimental system described in this study successfully tracked the collective movement patterns of the sea lice population in the horizontal plane during exposure to different light stimuli emitted from alternating zones in the system (Nordtug et al., submitted). The total number of detected individuals observed during the experiment decreased by almost 50% during a 10-min period (Fig. 2A). This decrease caused a nearly 100% increase in standard error (Fig. 2B), the decrease in detected lice was caused by the background correction algorithm not detecting individuals that had been stationary for longer than 5 s. However, this error did not seem to affect the position tracking significantly (Fig. 2B,D). The cause of the apparent reduction in swimming activity is not clear. It could be a result of the copepodites adapting to the stimuli, or it could be an artefact from changes in swimming patterns as the copepodites met the physical boundary (polycarbonate cylinder) in front of the light source. Exhaustion is less likely since it is reported that individual copepodites can maintain swimming speeds of more than 5 mm/s for at least 90 min (Fields et al., 2018). This issue could have been resolved by using a higher camera resolution yielding more pixels per individual; a higher camera resolution would better distinguish the lice from the image sensor- and background noise. This would open the possibility of removing the background correction algorithm, making it possible to use the initial frame for background correction instead. On the other hand, in this study we generated 10 MB of data per second and 72 GB of data per experimental run. Using a camera with higher resolution would certainly yield more robust data, as well as enabling the possibility of studying even smaller organisms, but this would naturally also generate larger data sets requiring higher storage capacity and more computational processing power.

Compared to other experimental systems, such as the Y-tube setup (Mordue and Birkett, 2009) where the organisms can only move in two directions, the system described for this study offers fewer restrictions as the lice are allowed to move freely in all directions, including up and down in the water column, within the boundaries of the inner peripheral of the polycarbonate cylinder. The system was not able to detect and record vertical movements as upward swimming or passive sinking, they were recognized as stationary particles and were undetected by the algorithm. One option to fix this would be to implement a three-dimensional (3D) tracking system like a stereo vision system; however, this would significantly complicate the data processing, without guaranteeing better results as we were already pushing the limits due to the size of the organisms (800 µm×200 µm), covering less than four pixels on average.

The system did not take the light refractions into account due to the air–water transition, which impacted the position and velocity measurements. However, as the camera was placed dead center in the observational area with the same geometrical distance from all zones (P1 to P4); there was no bias towards any of the physical cues. In addition, the light refraction error could be reduced by placing the calibration frame in the middle of the water column during the calibration routine.

This system facilitated a significantly larger observational volume, advantageously and completely without blind zones, compared to other experimental set-ups (Flamarique et al., 2000; Solvang and Hagemann, 2018). Moving forward, this system could be further improved by hardware upgrades to increase image resolution, image acquisition frequency and tracking accuracy down to an individual level.

In summary, we successfully utilized this system to identify behavioral responses of *L. salmonis* copepodites to light of different wavelengths and intensities. The system could further be used to study behavioral responses of different developmental stages of sea lice (nauplii I & II), or other zooplankton to light or other physical or chemical cues.

## MATERIALS AND METHODS

To study the behavioral responses to physical cues (in this case light) in *L. salmonis*, isolating the system from any additional stimuli was paramount. We chose to use a camera system combined with a near infrared (NIR) light source, emitting wavelengths invisible to the sea lice, to allow the system to automatically sample position data without intervening with the experiment. In machine vision systems in general, keeping the interference of outside elements (i.e. reflections, sensor noise and foreign objects) to a minimum is essential for creating efficient and robust algorithms based on classical image processing for detecting and tracking objects.

### Experimental setup

**Fig. 1.**
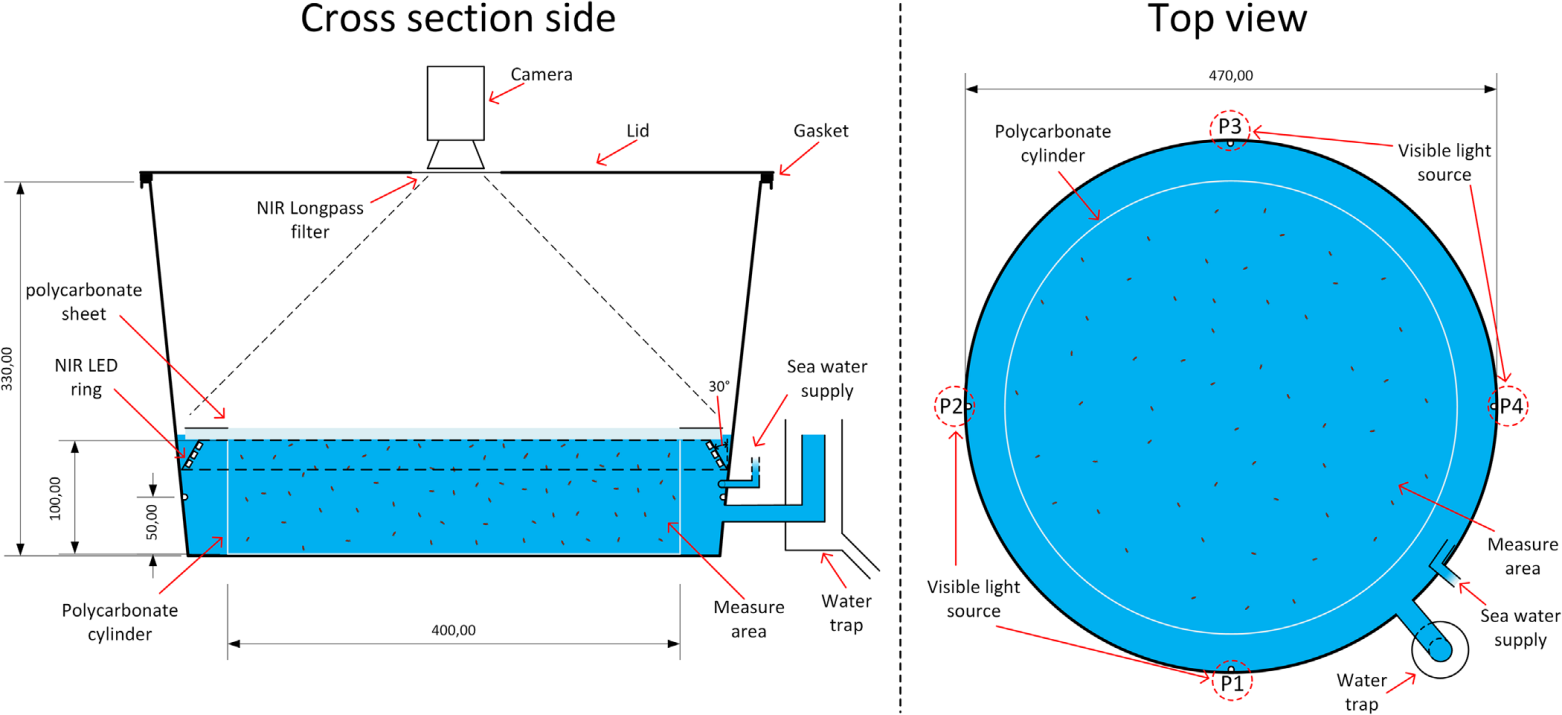
**A sketch of an experimental setup for studying lice population behavior within the ‘Measure area’ using ‘Visible light sources’ as stimuli.** Sampling lice position data with a ‘camera’ utilizing a ‘NIR light’ source for camera lighting and a ‘water trap’ system for water circulation and NIR light source cooling.

The experimental system (Fig. 1) was constructed inside a black high-density polyethylene (HDPE) container (330 mm height, 470 mm diameter, 60 l) having good light absorption property to minimize light reflection from the walls of the container. A lid with a soft gasket was placed on top of the container to isolate the interior from light pollution. A clear polycarbonate resin thermoplastic cylinder (100 mm height, 400 mm diameter, polycarbonate), slightly smaller than the bottom peripheral area of the container, was placed at the bottom of the container in order to retain the lice within the camera field of view (FOV) and to keep the lice away from camera blind spots such as the NIR light source. The observational volume of the system is 12.5 l.

A NIR LED-strip (SMD5050-600-IR, 850 nm, IP68, 28.8 W/m, LEDLightsWorld, USA) was mounted at a 30° angle along the HDPE container wall to obtain a uniform illumination of the whole measurement area. The NIR LED-strip (85 W in total) produced enough light to acquire frames with the lens aperture set to f/3, which gave a focal depth covering the entire water column in the measurement area (100 mm). To avoid temperature increasing inside the measurement area due to heat transfer from the NIR LED's, a constant flow of cooled seawater was circulated between the polycarbonate cylinder and the HDPE container with the IR LED-strip. An adjustable water trap was installed to set the cooling water level. The LED's (T1-3/4″, RGB, Cyan, UV, 20 mA) used for inducing different light stimuli were mounted through the wall of the outer HDPE container in direct contact with the cooling water 5 cm below the water surface (2 cm below the NIR LED-strip). The light intensity was controlled using pulse width modulation (PWM).

A Point Grey Grasshopper 3 camera (2448×2048, Mono chrome, FLIR, USA) equipped with a wide FOV TECHSPEC lens (4 mm, 84° FOV, Edmund Optics, USA) was setup with an acquisition rate of two frames per second. The high-resolution camera had a theoretical smallest particle detection limit (PDL) of approximately 200 µm (400 mm/2048 pixels), which allowed us to identify individual copepodites which are about 800 µm long and 200 µm wide. The camera sensor's (Sony IMX250) spectral range was 400–1000 nm, which made it compatible with the 850 nm NIR light source. However, for this design, a filter (Wratten 2, 800 nm, NIR, long pass filter, Kodak, USA) was placed in front of the camera lens to filter out visible light.

### Data processing

Frames acquired by the camera were stored locally on a computer during the experiments and post-processed using an algorithm written in Python 3.6 using OpenCV 3.4. Each frame was calibrated using the OpenCV (v3.4) implementation of Zhang's “A flexible new technique for camera calibration” (Zhang, 2000) before being processed. The data processing algorithm was designed to detect moving particles from one frame to another, removing particles that have been stationary for more than 5 s, extracting only moving particles for further processing (Table 1; Movie 1).

**Table 1.**
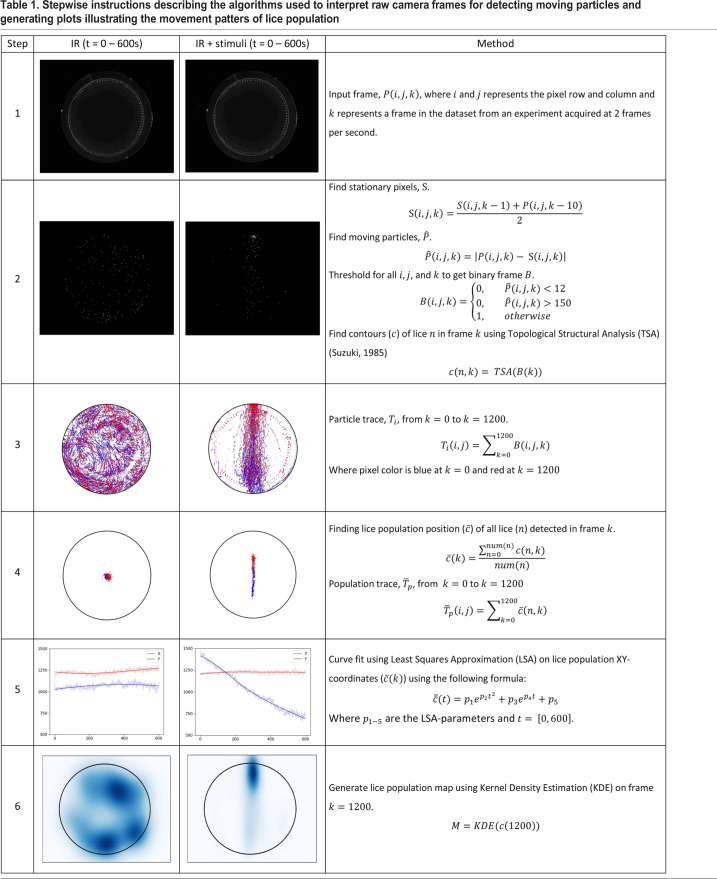
**Stepwise instructions describing the algorithms used to interpret raw camera frames for detecting moving particles and generating plots illustrating the movement patters of lice population**

Due to the perspective of the camera, the relative diameter (in pixels) is greater on top than at the bottom of the polycarbonate cylinder. Without being able to track the vertical position of the lice it is impossible to find a horizontal pixels-to-mm parameter that is suitable for the entire water volume. This issue was simplified by assuming the mean lice population position is in the middle of the water column due to the position of the visible light LED's. The polycarbonate cylinder diameter was measured in the middle of the water column both in relative (*l_m_*=1730 pixels) and in absolute measurements (L=400 mm) in order to calculate the pixels-to-mm parameter (Eqn 1).(1)
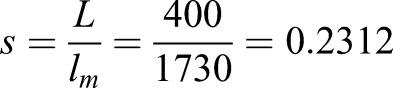
The population velocity was found by taking the derivated population position 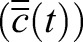 and multiplying by the pixels-to-mm parameter (Eqn 2).(2)
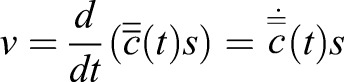
However, one must correct for the perspective effect, where a particle seems to move slower when the horizontal distance from the camera increases. The equation uses the population position (*x*, *y* and *z*) to correct the velocity (*v*) of the moving population (*c*(*n*, *k*)) so that it appear as measured in front of the lens (*x*=200 and *y*=200, Eqn 3).(3)
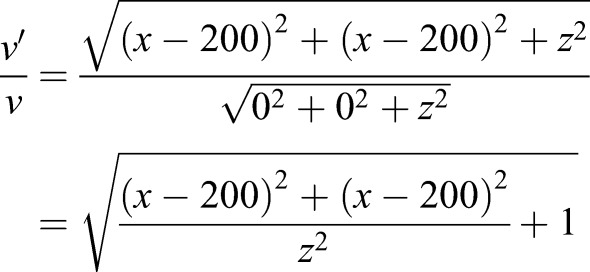
The function for calculating the corrected velocity (*v*^′^) was found by solving the expression in term of ‘*v*^′^’, and setting ‘*z*’ to the length from the horizontal plane (the middle of the water column) to the lens (280 mm, Eqn 4).(4)
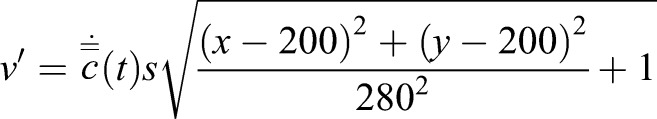
As stated, both position and velocity measurements were calculated with the assumption that the mean lice population position is in the middle of the water column. Should the population deviate from this assumption the potential position error (*e*) was calculated by measuring the relative height (*h*=216 pixels) from top- to bottom edge of the cylinder (Eqn 5).(5)
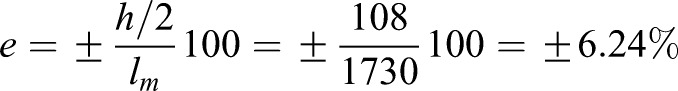


### Experiments

**Fig. 2.**
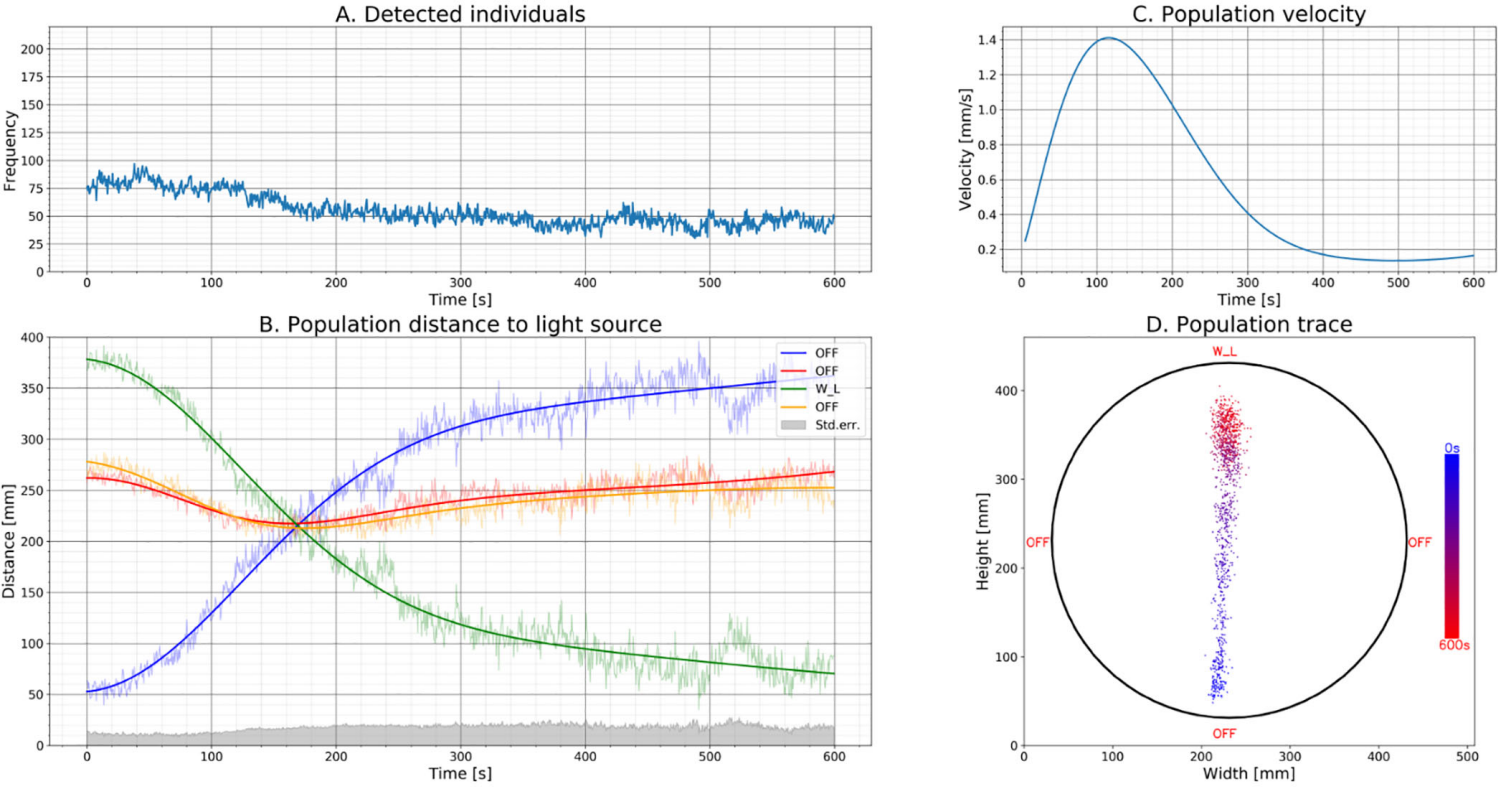
**Plot illustrating population behavior of *L. salmonis* copepodites when exposed to a white LED point source.** Plot A illustrates the number of detected lice (frequency) over time (s). Plot B illustrates the lice population distance to the different light sources over time presented as both raw population position and curve fit using least square approximation. Plot C illustrates the population velocity over time, where the velocity is derived from curve fitted population position data. Plot D illustrates the population migration in the measurement area at t=0 (blue) to t=600 (red). ‘W_L' indicates that the light source is set to ‘white light' at ‘low intensity' (L=1.5×10^−3^ μmol m^−2^ s^−1^ in the center of the arena), and ‘OFF’ indicates that the light source is switched off.

Salmon louse (*L. salmonis*) copepodites were acquired from The Industrial Aquatic Laboratory (ILAB, 5008 Bergen, Norway). Before each experimental run, living copepodites (*n*=200) were counted and checked for vitality before being placed in the experimental system. The copepodite stage is the infective developmental stage, which is the last of the three free swimming stages (Hamre et al., 2013) that depends solely on endogenous energy reserves up until they find and successfully attach to a host. Hence, we did not add any food items into the experimental system nor feed the copepodites kept in stock. Each experimental run was pre-programmed using an Arduino to control the different LEDs placed at zone P1–P4 (Fig. 1). One RGB-, one Cyan- and one UV LED were positioned at each zone. The LEDs were pre-set to emit light at different combinations of both intensity and frequency from alternating zones, whereof each pre-set illumination lasted for 10 min. The different light combinations tested during one experimental run lasted for a period of 120 min (12 presets) in total. The camera acquired two frames per second, generating 14,400 frames per experiment. Obtained data was analyzed using the algorithms explained in Table 1. A subset of the results is presented in Fig. 2, to illustrate how the behavioral responses of the *L. salmonis* copepodites to light were outlined and parametrized, while the results are shown in its entirety in Nordtug et al. (submitted).

## Supplementary Material

Supplementary information

## References

[BIO050724C1] Aarseth K. A. and Schram T. A. (1999). Wavelength-specific behaviour in Lepeophtheirus salmonis and Calanus finmarchicus to ultraviolet and visible light in laboratory experiments (Crustacea: Copepoda). *Mar. Ecol. Prog. Ser.* 186, 211-217. 10.3354/meps186211

[BIO050724C2] Amundrud T. L. and Murray A. G. (2009). Modelling sea lice dispersion under varying environmental forcing in a Scottish sea loch. *J. Fish Dis.* 32, 27-44. 10.1111/j.1365-2761.2008.00980.x 19245629

[BIO050724C3] Bailey R. J. E., Birkett M. A., Ingvarsdóttir A., Mordue A. J., Mordue W., O'Shea B., Pickett J. A. and Wadhams L. J. (2006). The role of semiochemicals in host location and non-host avoidance by salmon louse (Lepeophtheirus salmonis) copepodids. *Can. J. Fish. Aquat. Sci.* 63, 448-456. 10.1139/f05-231

[BIO050724C4] Costello M. J. (2009). The global economic cost of sea lice to the salmonid farming industry. *J. Fish Dis.* 32, 115-118. 10.1111/j.1365-2761.2008.01011.x 19245636

[BIO050724C5] Fields D. M., Skiftesvik A. B. and Browman H. I. (2018). Behavioural responses of infective-stage copepodids of the salmon louse (Lepeophtheirus salmonis, Copepoda: Caligidae) to host-related sensory cues. *J. Fish Dis.* 41, 875-884. 10.1111/jfd.12690 28921570

[BIO050724C6] Flamarique I. N., Browman H. I., Belanger M. and Boxaspen K. (2000). Ontogenetic changes in visual sensitivity of the parasitic salmon louse Lepeophtheirus salmonis. *J. Exp. Biol.* 203, 1649-1657. 10804155 10.1242/jeb.203.11.1649

[BIO050724C7] Gravil H. R. (1996). Studies on the biology and ecology of the free swimming larval stages of Lepeophtheirus salmonis (Kroyer, 1838) and Caligus elongatus Nordmann, 1832 (Copepoda: Caligidae) http://hdl.handle.net/1893/2380

[BIO050724C9] Heuch P. A., Parsons A. and Boxaspen K. (1995). Diel vertical migration: a possible host-finding mechanism in salmon louse (Lepeophtheirus salmonis) copepodids? *Can. J. Fish. Aquat. Sci.* 52, 681-689. 10.1139/f95-069

[BIO050724C10] Heuch P. A., Parsons A. and Boxaspen K. (1996). Diel vertical migration: a possible host-finding mechanism in salmon louse (Lepeophtheirus salmonis) copepodids? *Oceanogr. Literature Rev.* 52, 681-689. 10.1139/f95-069

[BIO050724C11] Hevrøy E. M., Boxaspen K., Oppedal F., Taranger G. L. and Holm J. C. (2003). The effect of artificial light treatment and depth on the infestation of the sea louse Lepeophtheirus salmonis on Atlantic salmon (Salmo salar L.) culture. *Aquaculture* 220, 1-14. 10.1016/S0044-8486(02)00189-8

[BIO050724C19] Johnson S. C. and Albright. L. J. ‘The developmental stages of Lepeophtheirus salmonis (Krøyer, 1837) (Copepoda: Caligidae).’ *Canadian Journal of Zoology* 69, 929-950. 10.1139/z91-138

[BIO050724C12] Kristoffersen A. B., Jimenez D., Viljugrein H., Grøntvedt R., Stien A. and Jansen P. A. (2014). Large scale modelling of salmon lice (Lepeophtheirus salmonis) infection pressure based on lice monitoring data from Norwegian salmonid farms. *Epidemics* 9, 31-39. 10.1016/j.epidem.2014.09.007 25480132

[BIO050724C13] Mordue A. J. and Birkett M. A. (2009). A review of host finding behaviour in the parasitic sea louse, Lepeophtheirus salmonis (Caligidae: Copepoda). *J. Fish Dis.* 32, 3-13. 10.1111/j.1365-2761.2008.01004.x 19245627

[BIO050724C14] Myksvoll M. S., Sandvik A. D., Albretsen J., Asplin L., Johnsen I. A., Karlsen Ø., Kristensen N. M., Melsom A., Skardhamar J. and Ådlandsvik B. (2018). Evaluation of a national operational salmon lice monitoring system—From physics to fish. *PLoS ONE* 13, e0201338 10.1371/journal.pone.0201338 30063759 PMC6067748

[BIO050724C15] Nordtug T., Kvæstad B. and Hagemann A. (submitted). Responses and preferences of salmon louse (Lepeophtheirus salmonis Krøyer 1836) copepodites to underwater artificial light sources.

[BIO050724C16] Solvang T. and Hagemann A. (2018). A machine vision system for zooplankton behavioural studies: a case study on the phototactic behaviour of sea lice (Lepeophtheirus salmonis) during sound and ultrasound stimuli. *J. Exp. Biol.* 221, jeb183277 10.1242/jeb.183277 30002094

[BIO050724C17] Suzuki S. and be K. A. (1985). Topological structural analysis of digitized binary images by border following. *Comput. Vis. Graphics Image Process.* 30, 32-46. 10.1016/0734-189X(85)90016-7

[BIO050724C18] Zhang Z. (2000). A flexible new technique for camera calibration. *IEEE Trans. Pattern Anal. Mach. Intell.* 22, 1330-1334. 10.1109/34.888718

