# Supplementary figures and images for: A machine vision system for tracking population behavior of zooplankton in small-scale experiments: a case study on salmon lice (Lepeophtheirus salmonis Krøyer, 1838) copepodite population responses to different light stimuli
Source: Biol Open. 2020 Jun 25;9(6):bio050724. doi: 10.1242/bio.050724 (PMC7328005; doi:10.1242/bio.050724)

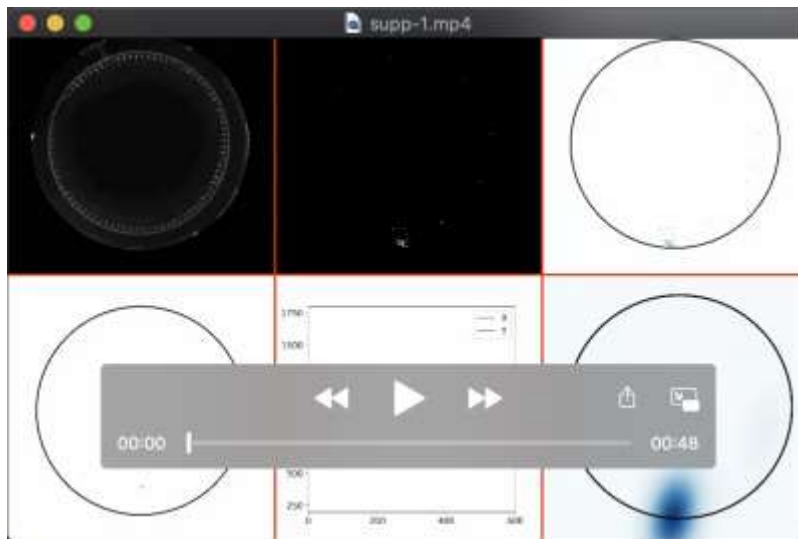

Movie 1

Supplement: Supplementary information [file biolopen-9-050724-s1.pdf]
